# Factors associated with patient, and diagnostic delays in Chinese TB patients: a systematic review and meta-analysis

**DOI:** 10.1186/1741-7015-11-156

**Published:** 2013-07-02

**Authors:** Ying Li, John Ehiri, Shenglan Tang, Daikun Li, Yongqiao Bian, Hui Lin, Caitlin Marshall, Jia Cao

**Affiliations:** 1 Department of Social Medicine and Health Service Management, College of Preventive Medicine, Third Military Medical University, No. 30 Gaotanyan Road, Shapingba District, Chongqing, China; 2 Division of Health Promotion Sciences, Mel and Enid Zuckerman College of Public Health, University of Arizona, 1295 N. Martin Avenue, Tucson, AZ, USA; 3 Duke Global Health Institute, Duke University, 310 Trent Drive, Durham, NC, USA; 4 Department of Laboratory Medicine, University-Town Hospital of Chongqing University of Medical Sciences, No. 55 Daxuecheng Road, Shapingba District, Chongqing, China; 5 Department of Epidemiology, College of Preventive Medicine, Third Military Medical University, No. 30 Gaotanyan Road, Shapingba District, Chongqing, China; 6 Key Lab of Medical Protection for Electromagnetic Radiation, Ministry of Education of China, College of Preventive Medicine, Third Military Medical University, No. 30 Gaotanyan Road, Shapingba District, Chongqing, China; 7 Toxicology institute, College of Preventive Medicine, Third Military Medical University, No. 30 Gaotanyan Road, Shapingba district, Chongqing 400038, China

**Keywords:** Tuberculosis, Patient delay, Diagnosis delay, Risk factors

## Abstract

**Background:**

Delay in seeking care is a major impediment to effective management of tuberculosis (TB) in China. To elucidate factors that underpin patient and diagnostic delays in TB management, we conducted a systematic review and meta-analysis of factors that are associated with delays in TB care-seeking and diagnosis in the country.

**Methods:**

This review was prepared following standard procedures of the Cochrane Collaboration and the Preferred Reporting Items for Systematic Reviews and Meta-Analyses statement and checklist. Relevant studies published up to November 2012 were identified from three major international and Chinese literature databases: Medline/PubMed, EMBASE and CNKI (China National Knowledge Infrastructure).

**Results:**

We included 29 studies involving 38,947 patients from 17 provinces in China. Qualitative analysis showed that key individual level determinants of delays included socio-demographic and economic factors, mostly poverty, rural residence, lack of health insurance, lower educational attainment, stigma and poor knowledge of TB. Health facility determinants included limited availability of resources to perform prompt diagnosis, lack of qualified health workers and geographical barriers.

Quantitative meta-analysis indicated that living in rural areas was a risk factor for patient delays (pooled odds ratio (OR) (95% confidence interval (CI)): 1.79 (1.62, 1.98)) and diagnostic delays (pooled OR (95% CI): 1.40 (1.23, 1.59)). Female patients had higher risk of patient delay (pooled OR (95% CI): 1.94 (1.13, 3.33)). Low educational attainment (primary school and below) was also a risk factor for patient delay (pooled OR (95% CI): 2.14 (1.03, 4.47)). The practice of seeking care first from Traditional Chinese Medicine (TMC) providers was also identified as a risk factor for diagnostic delay (pooled OR (95% CI): 5.75 (3.03, 10.94)).

**Conclusion:**

Patient and diagnostic delays in TB care are mediated by individual and health facility factors. Population-based interventions that seek to reduce TB stigma and raise awareness about the benefits of early diagnosis and prompt treatment are needed. Policies that remove patients’ financial barriers in access to TB care, and integration of the informal care sector into TB control in urban and rural settings are central factors in TB control.

## Background

Although progress has been made in global tuberculosis (TB) control [1], the disease remains one of the most intractable health challenges in low- and middle-income countries. Globally, TB remains the second leading cause of death from infectious diseases (after HIV/AIDS) [1]. Over the past five years, there has been a marked increase in the number of TB patients diagnosed with multi-drug resistant TB (MDR-TB) [1]. China has the world’s second largest tuberculosis epidemic and has contributed significantly to this MDR-TB increase. Results of the 5th National TB Survey in China in 2010 showed an estimated 8.28 million cases of pulmonary TB (PTB) in the country from 2001 to 2010 [2]. In spite of concerted international and national efforts to address TB in the country, annual prevalence (per 100,000 people) declined only minimally from 466 in 2000 to 459 in 2010 [2]. Similarly, incidence of MDR-TB among PTB cases has remained unchanged in the past decade, and resistance to first-line drugs among newly diagnosed TB patients rose from 34.2% in 2007/2008 [3] to 36.9% in 2010 [2].

Limited knowledge about signs and symptoms of TB, poor health seeking behavior, and poor diagnosis and disease management in health facilities result in delays in TB diagnosis and treatment, which in turn, increase the risk of TB transmission and the development of MDR-TB [4]. Available evidence shows that only 47% of Chinese patients with TB symptoms seek healthcare in a timely fashion and only 59% comply with prescribed treatment [2].

Early diagnosis and prompt treatment are the core objectives of an effective national TB control program [5]. Persons who experience signs and symptoms of TB should report promptly for diagnosis, and should, if found to have TB, commence treatment immediately [5]. Unfortunately, this process can be disrupted by delay in deciding to visit a TB treatment facility [6-8], delay in obtaining diagnosis [9] and delay in receiving treatment [10]. These types of delays are reported to be common in many low- and middle-income countries which have a high TB burden [8,11-17].

Although reviews conducted on TB care-seeking behavior in sub-Saharan Africa and elsewhere [18,19] suggest that the epidemiology and control of TB are highly associated with socioeconomic and cultural factors, China has a unique social, economic and cultural context. Thus, understanding the sociocultural milieu of delays in TB management in China could help to stem the high burden of TB in the country. A few studies have been conducted to explore factors that influence delays in TB care-seeking, diagnosis and treatment in China. Unfortunately, results of these studies have been largely inconclusive and inconsistent [20-23]. The objective of this review is to critically summarize available literature on factors that underpin delays in pulmonary TB care-seeking, and diagnosis in China. The results could contribute to current efforts to develop a sustainable and effective TB control program in the country.

## Methods

### Definition

According to the Chinese Ministry of Health (MOHC) [24], “patient delay” is defined as a time interval of more than two weeks between the onset of TB symptoms and the patient’s first presentation to a health facility. The Ministry also defines “diagnostic delay” as a time interval of more than two weeks between a patient’s first visit to a health facility and the receipt of TB diagnosis.

### Search strategy

The review was prepared following standard procedures of the Cochrane Collaboration [25] and the Preferred Reporting Items for Systematic Reviews and Meta-Analyses (PRISMA) statement and checklist [26] (see Additional file 1). To identify eligible studies on factors associated with TB patient and diagnostic delays published up to November 2012, three major databases, Medline/PubMed, EMBASE and CNKI (China National Knowledge Infrastructure), were searched. We used a mixture of free text and index terms to maximize retrieval of potentially relevant studies. The following terms were used for the PubMed search: “pulmonary tuberculosis and diagnostic delay in China”, “pulmonary tuberculosis and health system delay in China”, “pulmonary tuberculosis and detection delay in China”, “pulmonary tuberculosis and patient delay in China”, “pulmonary tuberculosis and health provider delay in China”, “pulmonary tuberculosis and doctor delay in China”, “pulmonary tuberculosis and identification delay in China”. Medline subject search terms included: “tuberculosis, pulmonary”[Mesh]) AND “diagnosis”[Mesh]) AND “China”[Mesh] or “tuberculosis, pulmonary”[Mesh]) AND “patient acceptance of health care”[Mesh]) AND “China”[Mesh]. Searches in Medline/Pubmed were limited to full-text, humans, and English. The search terms used in EMBASE were ‘lung tuberculosis’/exp AND ‘early diagnosis’/exp AND ‘risk factor’/exp, ‘lung tuberculosis’/exp AND ‘patient’/exp AND ‘delayed diagnosis’/exp, ‘lung tuberculosis’/exp AND ‘delayed diagnosis’/exp AND ‘risk factor’/exp, ‘lung tuberculosis’/exp AND ‘patient’/exp AND ‘patient referral’/exp AND ‘risk factor’/exp, ‘lung tuberculosis’/exp AND ‘health care facility’/exp AND ‘patient referral’/exp AND ‘risk factor’/exp. CNKI is a key national e-publishing project that can be used for searching articles published in 8,200 Chinese journals. It was utilized to search for articles in Chinese. The terms and concepts searched included “patient delay”, “diagnostic delay”, “risk factors for patient delay” and “risk factors for diagnostic delay”. We sought unpublished data from the gray literature through Google and Google Scholar searches. We also hand-searched reference lists of identified articles. Two reviewers (JE and YL) conducted the literature searches.

### Inclusion/exclusion criteria

(1) Types of studies: Case-control, cross-sectional and cohort studies were included.

(2) Participants: Patients with pulmonary tuberculosis (including smear positive or negative patients, newly diagnosed cases and those undergoing retreatment).

(3) Exposure variables: We collected data on the following:

(i) Demographic characteristics: marital status (classified as married or living with partner, single, widowed, divorced), age (grouped into 60 years and older, and less than 60 years old), education level (categorized into primary school and below, middle school and above), residence (categorized into rural and urban).

(i) Risk behavior/lifestyle: smoking and alcohol consumption (categorized into smoker, non-smoker, alcohol drinker and non-drinker).

(i) Health systems factors: health facilities (categorized into non-TB health facilities and TB health facilities), health resource related to TB diagnosis, ability of health providers and traffic or geographical barriers.

(4) Outcomes measures: TB patient delay and TB diagnosis delay.

When there was evidence of multiple publications of the same study over time, only the article with a full report was included. Reports on TB suspects, extra-pulmonary TB, HIV infected TB patients, qualitative studies (data collected by qualitative research methods) and studies that focus on PTB patients in Hong Kong, Macao and Taiwan were excluded.

### Study selection

Application of inclusion and exclusion criteria to identified studies was done by two reviewers (DL and YB). Both reviewers independently screened titles and abstracts of identified studies to assess their eligibility for inclusion in the review, using an eligibility form based on the inclusion criteria. Where there were disagreements regarding eligibility of studies, all reviewers participated in the decision about inclusion by discussion and consensus.

### Quality assessment

We assessed the quality of case-control and cohort studies using the Newcastle-Ottawa Scale [27]. For case-control studies, we assessed the adequacy of case and control definition, representativeness of the cases, whether controls were derived from the same population as cases, comparability of cases and controls on the basis of design and analyses, ascertainment of exposure and non-response rates. For cohort studies, we assessed representativeness of the exposed cohort in the study setting, selection of non-exposed cohort, ascertainment of exposure, evidence that participants do not have outcome of interest at recruitment into the study, comparability of cohorts on the basis of design and analyses, outcome assessment and adequacy of follow-up [27]. After assessing the quality of each included study on the basis of these criteria, we assigned a composite quality score ranging from 0 (low) to 8 (high).

For cross-sectional studies, we used the guideline for critical appraisal of cross-sectional studies developed by the National Collaborating Center for Environmental Health [28], which was adapted from a combination of items from the Newcastle-Ottawa Scale [27], the Critical Appraisal Skills Program [29], Critical Appraisals by Elwood [30], and Aschengrau and Seage III [31]. Specifically, we assessed representativeness of the study participants, methods for ascertaining exposure; comparability of exposure groups (including unexposed) in terms of age, sex, socioeconomic status and response bias; determination and validation of outcomes; internal validity; and how confounding factors were assessed and addressed. After reviewing the quality of each included study on the basis of these criteria, we assigned a composite quality score that ranged from 0 (low) to 4 (high). Two reviewers (YL and JE) assessed study quality and reached a consensus grade for each included study.

### Data abstraction

Data from eligible studies were independently abstracted by two reviewers (BY and KL). Differences were resolved by consensus among all reviewers. Data extracted from each study included name of first author, year of publication, type of study design, place of study, type of participants (newly diagnosed/retreatment and smear positive/negative pulmonary TB patients), residence of participants (rural/urban), sampling size, outcome (patient delay/diagnostic delay), information used for comparing analyses (numbers of participants with/without exposure variables) and the main results were extracted for all included studies.

### Assessment of heterogeneity

Heterogeneity was evaluated using the Q test [32] and the I-squared statistic (I^2^ = 100% × (Q-df)/Q) [33]. For the Q test, a *P*-value of 0.10 or less was considered statistically significant, indicating marked heterogeneity among studies. Where the *P*-value was ≤0.10, we calculated I^2^, and studies with heterogeneity levels of ≤50% were deemed acceptable. Where heterogeneity was more than 50%, we conducted subgroup analysis to explore possible reasons for the heterogeneity. For subgroup analyses, the heterogeneity within groups was also tested, using the same statistical methods.

### Data synthesis

The first step of the data analysis involved a qualitative synthesis aimed at summarizing, comparing and contrasting the extracted data. Meta-analyses were then conducted to assess the association among patient delay, diagnostic delay, and gender, age, education, residence, healthcare seeking behaviors (TB health facility for first consultation or seeking care from Traditional Chinese Medicine (TMC) providers) and lifestyle factors (smoking and alcohol use). Only studies that adopted the definitions of patient and diagnostic delays proposed by the Office of TB Control in the MOHC [24] were included in the quantitative meta-analysis. We calculated pooled odds ratio (OR) separately for each factor, using RevMan 5.2 [25]. As noted earlier, we used the fixed effect model where the level of heterogeneity was acceptable (that is, *P* = >0.10, or *P* = ≤0.10, but I^2^ = ≤50%), and the random effects model was used where there was significant heterogeneity (that is, *P* = ≤0.10, but I^2^ = >50%). We assessed sources of heterogeneity by conducting sub-group analyses.

## Results

### Description of studies

**Figure 1:**
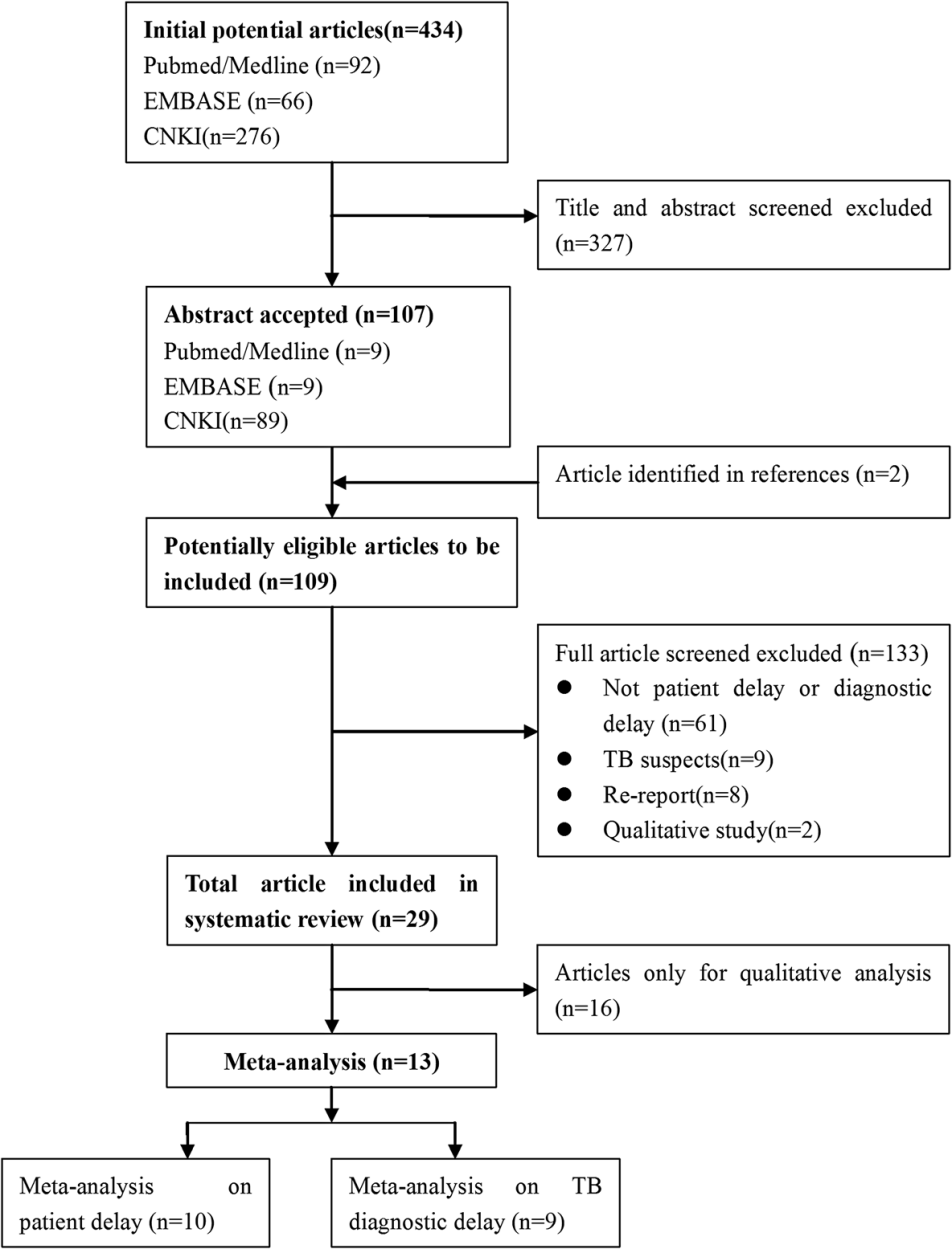
**Results of literature search.** This figure is a description of the full search process.

**Figure 2:**
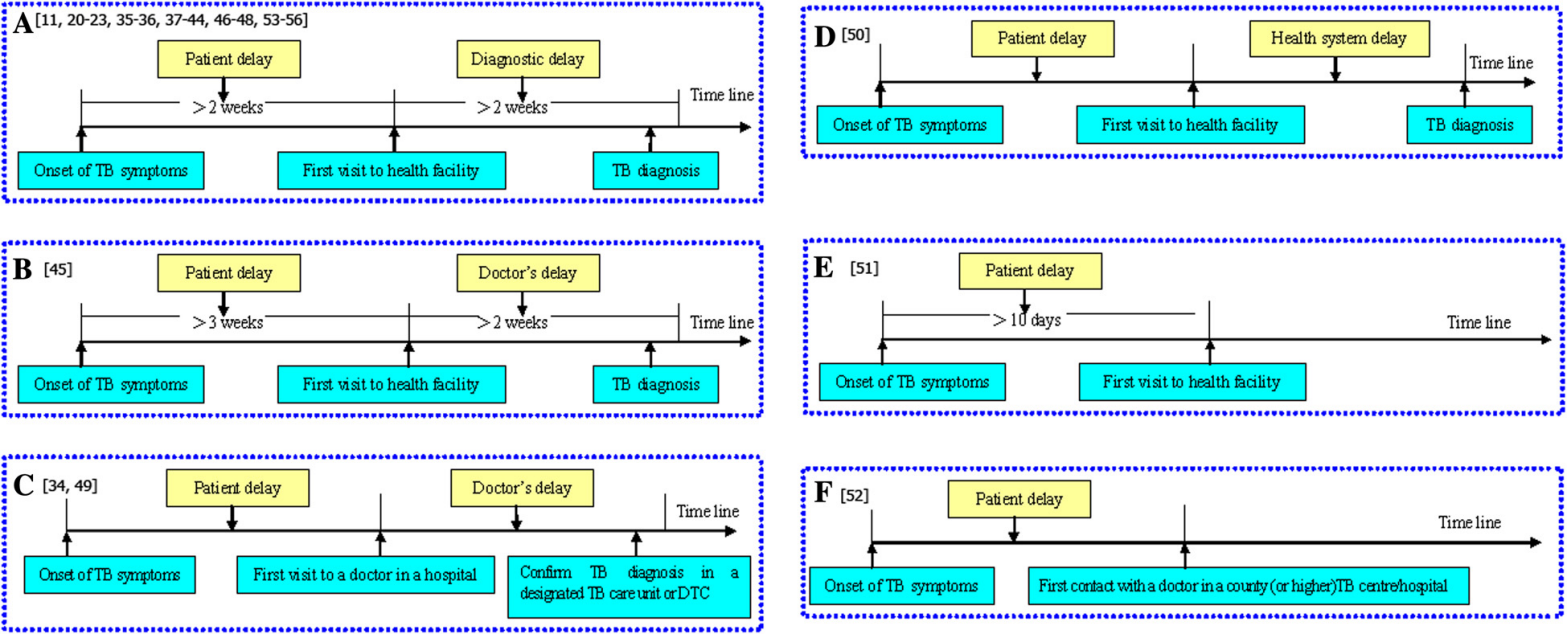
**This figure presents different definitions of delays used in studies on patient delay and diagnostic delay in mainland China. A**: Patient delay refers to the time from the onset of TB symptoms to first visit to health facility is greater than 2 weeks. Diagnostic delay refers to the time from the first visit to health facility to TB diagnosis is greater than 2 weeks. **B**: Patient delay refers to the time from the onset of TB symptoms to first visit to health facility is greater than 21days. Diagnostic delay refers to the time from the first visit to health facility to TB diagnosis is greater than 2 weeks. **C**: Patient delay refers to defined as the duration from the onset of symptoms to the first visit to a doctor in a hospital. Doctor’s delay was defined as the duration from the first hospital visit to a confirmed TB diagnosis in the designated DTC or designated TB Care Unit. **D**: Patient delay was defined as the time (in days) from the onset of symptoms to first seeking care at a health facility. Health system delay as the time (in days) from first seeking care at a health facility (including village clinics) to diagnosis. **E**: Delayed initial health-seeking was defined as duration in excess of the median duration (10 days) (including hospital, TB dispensary, clinic, health center, etc.). **F**: Patient delay is defined as the time from the onset of TB symptoms to the first contact with a doctor in a county (or higher) TB center/ hospital.

Figure 1 presents an illustration of the search output. Altogether, the initial search yielded 434 potentially relevant articles. Only 29 studies [14,20-23,34-57] met the criteria for inclusion in the review which involved 38,947 pulmonary TB patients from 17 provinces (Table 1). The included studies comprised 27 cross-sectional studies, 1 cohort, and 1 case-control study. A majority of the studies (16 out of 29) included newly diagnosed smear-positive PTB patients. The studies applied different definitions of patient and diagnostic delays (Figure 2). However, a majority (23 of 29 studies) adopted the definitions of MOHC (Figure 2A). All 29 studies were included in qualitative data synthesis. However, only 13 cross-sectional studies [14,20-23,36,38-42,44] with 23,917 TB patients had sufficient data to qualify for inclusion in the meta-analysis.

**Table 1:** Studies included in systematic review and meta-analysis

**Studies**	**Type of study**	**Place of studies**	**Participants**	**Residence (rural/urban)**	**Sample size**	**Outcome**	**Risk factors**
Zhang 2006 [14]	CS	Hunan Province	New SP PTB	Rural	318	DD (>2 weeks)	PD: No haemoptysis (OR (95% CI): 0.119 (0.041, 0.344)), distance to health facility (OR (95% CI): 24.73 (6. 872, 58. 56)), seeking care from traditional medicine providers (OR (95% CI): 14.39 (4.379, 47.30)), poverty (OR (95% CI): 23.53 (8.389, 65.96))
						PD (>2 weeks)	DD: Being female (OR (95% CI): 3.06 (1.412, 6.645)), low education (OR (95% CI): 0.57 (0.370, 0.794)), limited access to TB education (OR (95% CI): 0.45 (0.218, 0.534)), seeking care from traditional medicine providers (OR (95% CI): 2.42 (1.057, 5.536)), seeking prescription from herbalist (OR (95% CI): 2.53 (1.261, 5.077)), and stigma (OR (95% CI): 14.65 (6.217, 31.78)).
Xi 2011 [20]	CS	Shandong Province	PTB	Rural	819	DD (>2 weeks)	DD: First consultation with traditional medicine providers
He 2009 [21]	CS	Hebei Province	New SP PTB	migrants from Rural	168	PD (>2 weeks)	PD: Low education level (*P* <0.05), lack of health insurance (*P* <0.01)
Huan 2007 [22]	CS	Guizhou Province	New PTB	Rural/urban	200	PD (>2 weeks)	PD: Distance to health facility;
						DD (>2 weeks)	DD: Poor equipment in health facility, health worker’s poor TB awareness and knowledge, poor referral
Wu 2008 [23]	CS	Anhui Province	New SP PTB	Rural/urban	148	PD (>2 weeks)	PD: Poor TB awareness
						DD (>2 weeks)	DD: Lack of equipment and qualified health workers in township hospital.
Wang 2007 [34]	CS	Shanghai	New SP/SN PTB	No description	222	PD was defined as the duration from the onset of symptoms to the first visit to a doctor in a hospital.	PD: Low income level (OR (95% CI): 3.859 (1.040, 14.314) for Level 1/level 4, 5.369 (1.717, 16.787) for Level 2/level 4), without Haemoptysis (OR (95% CI): 0.347 (0.127, 0.948))
Bai 2004 [35]	CS	Yunnan Province	New/re-treatment SP/SN PTB	Rural/urban	142	PD (>2 weeks)	PD: Being male (P = 0.037), with spouse (*P* = 0.010), no hemoptysis (*P* = 0.035);
						DD (>2 weeks)	DD: No night swear (*P* = 0.030) and chest pain (*P* = 0.042), respiratory difficulty (*P* = 0.029), no timely referring (*P* = 0.001).
Liao 2009 [36]	CS	Zhejiang Province	New SP PTB, elderly	Rural	210	PD (>2 weeks)	PD: Female (*P* = 0. 018), lack of TB knowledge (*P* = 0.003), Low access to health facility (*P* = 0.009)
Yang 2010 [37]	CS	Hujian, Henan, Liaoling, Xinjiang Province	PTB	Rural	888	PD (>2 weeks)	PD: Being female, the elderly, lack of TB knowledge, poverty and traffic difficulty
Zhou 2009 [38]	CS	Henan Province	New PTB	No description	102	PD (>2 weeks)	PD: Poor TB awareness; poverty, traffic difficulty;
						DD (>2 weeks)	DD: Consulting with traditional medicine providers first
Li 2002 [39]	CS	Hunan Province	New SP PTB	Rural/urban	3275	PD (>2 weeks)	PD: Being male (*P* = 0. 003), 30 to 44 years old (*P* = 0. 009), poor TB awareness, poverty, traffic difficulty;
						DD (>2 weeks)	DD: 30 to 44 years old (*P* = 0. 0001), poor TB knowledge, without smear test in county hospital
Shi 2006 [40]	CS	Liaolin, Hujian, Xingjiang, Henan Province	New SP PTB	No description	186	DD (>2 weeks)	DD: Consulting traditional medicine providers first, no resource for TB diagnosis
Yu 2007 [41]	CS	Anhui Province	New SP PTB	Rural/urban	17873	PD (>2 weeks)	PD: Being female (*P* <0.05), Rural area;
						DD (>2 weeks)	DD: Rural and diagnosed in non-TB hospital, no resource for TB diagnosis
Zhang 2011 [42]	CS	Jiangsu Province	New SP PTB	Rural/urban	276	DD (>2 weeks)	DD: Consulting with traditional medicine providers first, no resource for TB diagnosis
Cai 2007 [43]	CS	Gansu Province	New/retreatment SP/SN PTB	Rural	150	PD (>2 weeks)	PD: The elderly, female, low income, and education level, poor TB knowledge
Chai 2008 [44]	CS	Henan Province	New SP PTB	Rural/urban	200	PD (>3 weeks)	PD: Poor TB awareness, no severe symptoms, busy with work and poverty; stigma for the female
						DD (>2 weeks)	DD: Misdiagnosis, no severe symptoms
Tian 2001 [45]	CS	Shangdong Province	PTB, the youth	No description	400	PD (>2 weeks)	PD: Knowledge of TB control unit and TB awareness
						DD (>2 weeks)	DD: No haemoptysis (OR (95%): 0.52 (0.27, 0.98)), night swearing (OR (95%): 0.44 (0.23, 0.85)), chest pain (OR (95%): 2.15 (1.12, 4.15)).
Yang 2011 [46]	CS	Jiangsu Province	New SP PTB	No description	105	PD (>2 weeks)	PD: Poor TB awareness, low income level, distance to health facilities
Cao 2002 [47]	CS	Shangdong Province	PTB,	migrants from Rural	314	PD (>2 weeks)	PD: Busy at with work (working more than 6 days per week) (OR (95% CI):6. 70 (2. 80, 16.03)), no health insurance (OR (95% CI): 2.27 (1.07, 4.82)), distance to health facility (OR(95% CI): 2.13 (0.73, 6.18)), no hemoptysis (OR(95% CI): 0. 30 (0. 10, 0. 91)),
Geng 2010 [48]	CS	Shangdong Province	New SP PTB	Rural	200	PD was defined as the duration from the onset of cough to the first visit to any healthcare provider	PD: Poor TB awareness, low income level, lack of knowledge of TB control unit (*F* = 4.39, *P* <0.01)
Shi 2008 [49]	CS	Shangdong Province	New SP TB	Rural	312	PD was defined as the time (in days) from the onset of symptoms to first seeking care at a health facility	PD: Aged 40 to 59 (Adjusted HR (95% CI):0.34 (0.17, 0.69)), low education (Adjusted HR (95% CI): 1.91 (1.16, 3.14)), distance to health facility (Adjusted HR (95% CI): 1.04 (0.98, 1.11))
							DD: Being female (Adjusted HR (95% CI):0.63 (0.43,0.92)), Consulting with traditional medicine providers first (Adjusted HR (95% CI):1.14 (0.52, 2.51))
Cheng 2005 [50]	CS	Shanghai, Guangdong, Jiangsu Province	New PTB	migrants	323	PD (≥10 days)	PD: Busy with work (AOR (95% CI):1.61 (1.03, 2.51)), no hemoptysis (AOR (95% CI): 0.48 (0.28 ,0.85))
Li 2012 [51]	CS	Shanghai, Guangdong and Jiangsu	new PTB	migrant from rural	323	Patient delay ≥10 days	Average monthly working days ≥24, and without hemoptysis or bloody sputum
Lin 2008 [52]	Cohort	Yunnan Province	SP TB,	Rural	10356	PD (≥60 days)	PD: Geographical barrier, age >40, low income level (Adjusted HR (95%): 0.61 (0.57, 0.65), 0.78 (0.67, 0.90), 0.90 (0.85, 0.94)).
Zhang 2008 [53]	CC	Jiangsu Province	Newly SP PTB elderly	No description	102	PD (>2 weeks)	PD: Poor Tb awareness
						DD (>2 weeks)	DD: Misdiagnosis
Hou 2001 [54]	CS	Hubei Province	New SP PTB	No description	823	PD (>2 weeks)	PD: Poor TB awareness, and low income level
						DD (>2 weeks)	DD: Consulting with traditional medicine providers first
Liu 2000 [55]	CS	Hunan Province	SP PTB,	Rural/urban	290	PD (>2 weeks)	PD: Poor TB awareness and knowledge;
						DD (>2 weeks)	DD: Misdiagnosis, poor referral, not prescribing smear test
Li 2010 [56]	CS	Shandong Province	PTB, elderly	Rural/urban	322	PD (>2 weeks)	PD: 61 to 65 years old and lower income level
Lian 2003 [57]	CS	Guangzhou Province	Newly SP PTB	No description	117	PD (>2 weeks)	PD: Poor TB awareness, low income level, busy with work, traffic barrier.
						DD (>2 weeks)	DD: Consulting with traditional medicine providers first

Notes:

CS refers to cross-sectional study and CC refers to case-control study. SP refers to smear positive and SN refers to smear negative. PTB refers to pulmonary tuberculosis.

PD refers to patient delay and DD refers to diagnostic delay. *AOR* adjusted odds ratio, *CI* confidence interval, *HR* hazard ratio; *OR* odds ratio.

The quality assessment of studies indicated that eight cross-sectional studies [14,34,35,46,48-51] had a high quality score of 4 (out of 4). Eighteen cross-sectional studies had a score of 3, mainly because they did not control for confounding factors (Table 2). The cohort study [52] and case-control study [53] had high quality scores of 7 and 8, respectively, (out of 8) (Table 3).

**Table 2:** **Quality assessment of the cross-sectional studies included in systematic review and meta-analysis**※

**Studies**	**Type of study**	**A**	**B**	**C**	**D**	**Total score**
Zhang 2006 [14]	CS	1	1	1	1	4
Xi 2011 [20]	CS	1	1	0	1	3
He 2009 [21]	CS	1	1	0	1	3
Huan 2007 [22]	CS	1	1	0	1	3
Wu 2008 [23]	CS	1	1	0	1	3
Wang 2007 [34]	CS	1	1	1	1	4
Bai 2004 [35]	CS	1	1	1	1	4
Liao 2009 [36]	CS	1	1	0	1	3
Yang 2010 [37]	CS	1	1	0	1	3
Zhou 2009 [38]	CS	1	1	0	1	3
Li 2002 [39]	CS	1	1	0	1	3
Shi 2006 [40]	CS	1	1	0	1	3
Yu 2007 [41]	CS	1	1	0	1	3
Zhang 2011 [42]	CS	1	1	0	1	3
Cai 2007 [43]	CS	1	1	0	1	3
Chai 2008 [44]	CS	1	1	0	1	3
Tian 2001 [45]	CS	1	1	0	1	3
Yang 2011 [46]	CS	1	1	1	1	4
Cao 2002 [47]	CS	1	1	0	1	3
Geng 2010 [48]	CS	1	1	1	1	4
Shi 2008 [49]	CS	1	1	1	1	4
Cheng 2005 [50]	CS	1	1	1	1	4
Li 2012 [51]	CS	1	1	1	1	4
Hou 2001 [54]	CS	1	1	0	1	3
Liu 2000 [55]	CS	1	1	0	1	3
Li 2010 [56]	CS	1	1	0	1	3
Lian 2003 [57]	CS	1	1	0	1	3

CC refers to case-control study; CS refers to cross-sectional study. ※ A. representativeness of study participants, B. proper methods to ascertain exposure, C. comparability of comparing analysis groups and D. lower non-response bias.

**Table 3:** Quality assessment of cohort and case-control studies*

**Studies**	**Study design**	**A**				**B**		**C**			**Total score**
		**A1**	**A2**	**A3**	**A4**	**B1**	**B2**	**C1**	**C2**	**C3**	
Lin 2008 [52]	Cohort	1	1	1	1	1	—	1	1	0	7
Zhang 2008 [53]	CC	1	1	1	1	1	0	1	1	1	8

*Notes:

For cohort studies: A. selection of the study groups (A1 indicates exposed cohort truly representative, non-exposed cohort drawn from the same community, A3 right method for ascertainment of exposure, A4 outcome of interest not present at start the representativeness of controls); B. cohorts comparable (B1 control of HIV risk factors except IPV, B2 control of any additional factor); C. outcome measurement (C1 quality of outcome assessment, C2, follow-up long enough for outcomes to occur, C3 complete accounting for cohorts).

For case-control studies: A. selection of the study groups (A1 right case definition, A2 right controls definition, A3 the representativeness of the cases, A4 the representativeness of controls); B. comparability of the groups (B1 control of main confounders, B2 control of any additional factor); C. ascertainment of the exposure (C1 appropriate method of exposure ascertainment, C2 same method of exposure ascertainment for cases and controls, C3 same non-response rate of case and control groups).

1 indicates the study met the criteria; 0 indicates the study did not meet the criteria; A dash indicates fulfillment of the criteria could not be determined.

### Data synthesis

#### Summary of qualitative findings

##### Individual level factors

*Patient’s socio-demographic factors:* Although results on the association between gender, age and patient, and diagnostic delays were fairly inconsistent (Table 1), a majority of the studies reported that females were more likely than males to experience patient delay [20,22,42,44] and diagnostic delay [14,35,50]. A majority of the studies (18 of 29) demonstrated that several indicators of socioeconomic status (low educational attainment, rural residence, lack of health insurance, low income and inability to afford time off work) were risk factors for patient and diagnostic delays.

*Patient’s healthcare seeking behaviors:* Many studies reported the first visit to non-TB control health facilities, notably, traditional healer providers, was a risk factor for diagnostic delay [14,35,36,39,41,50,54].

*Patient’s TB knowledge/awareness:* Seventeen studies demonstrated that lack of TB knowledge (symptoms of TB, transmission mode of TB and TB control facilities) and poor TB awareness were risk factors for patient delay (Table 1). Evidence [40] also showed that TB stigma was associated with patient delay, and diagnostic delay (OR (95% CI): 14.65 (6.217, 31.78)) [14]. Only one study [35] analyzed the associations among smoking, alcohol use and patient/diagnostic delays. This study demonstrated no significant associations.

##### Health systems factors

Seventeen studies analyzed the relationship between health facility related factors and patient/diagnostic delays. It was found [40-42] that limitation in availability of resources to perform prompt diagnosis (for example, lack of facilities for X-rays and sputum test at village and township level health facilities) was a risk factor for TB diagnostic delay. Shortage of trained health providers at TB control facilities (for example, county TB dispensary and designated county hospitals for TB care), and geographical barriers were important causes of TB diagnostic delay [57], as well as factors related to the health facility staff (health workers’ inadequate TB knowledge [22,38], inability to prescribe smear test for TB suspects [23,39,55], inability to refer TB suspects to county TB dispensaries or designated hospitals for TB care [21,38,55] and misdiagnosis [45,53,55,57]).

##### Summary of findings from meta-analysis

**Figure 3:**
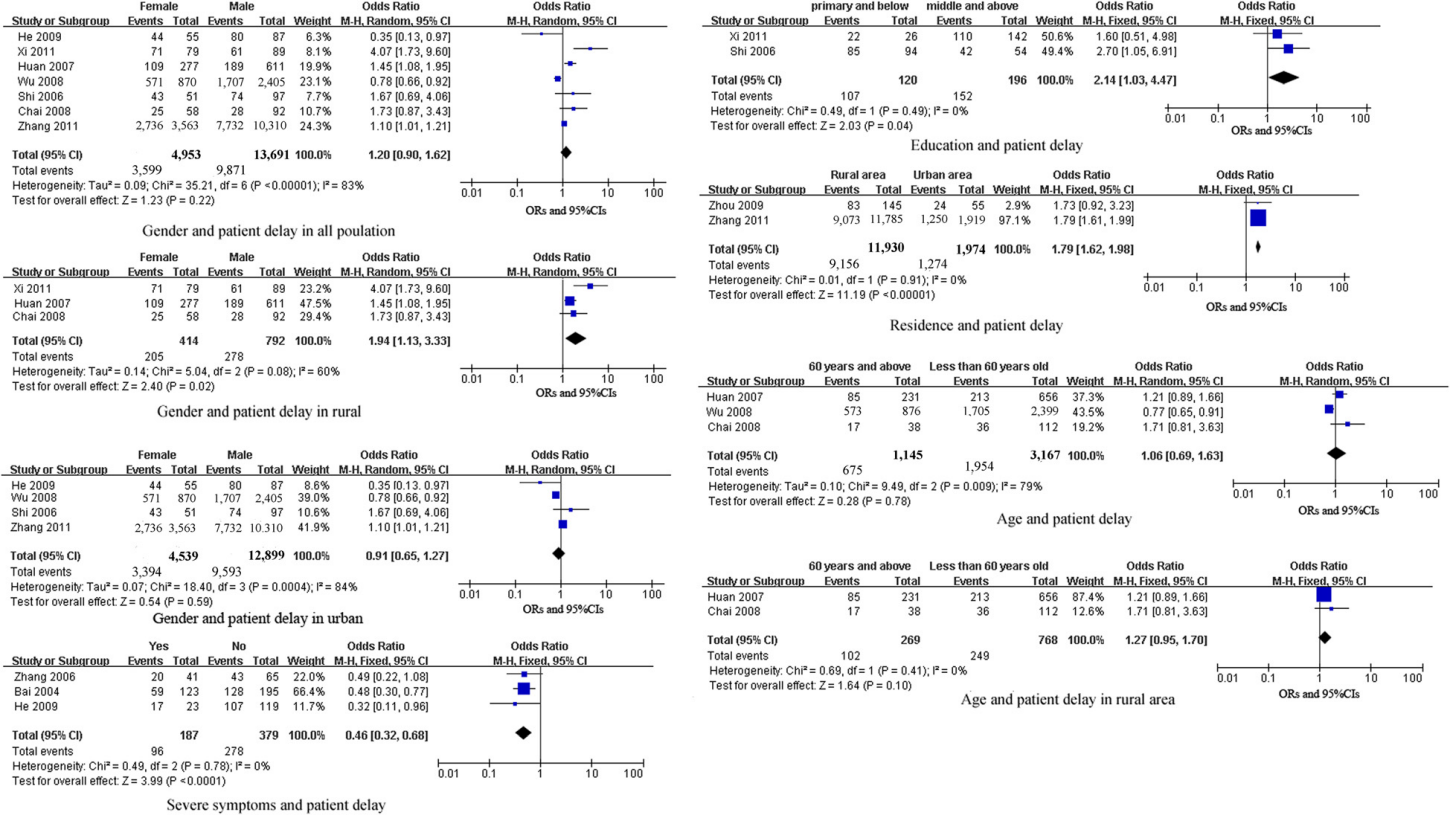
**Forest plots of meta-analysis of factors associated with patient delay.** This figure shows forest plots for the meta-analysis of factors associated with patient delay. OR and 99% CI for each factor are given.

**Figure 4:**
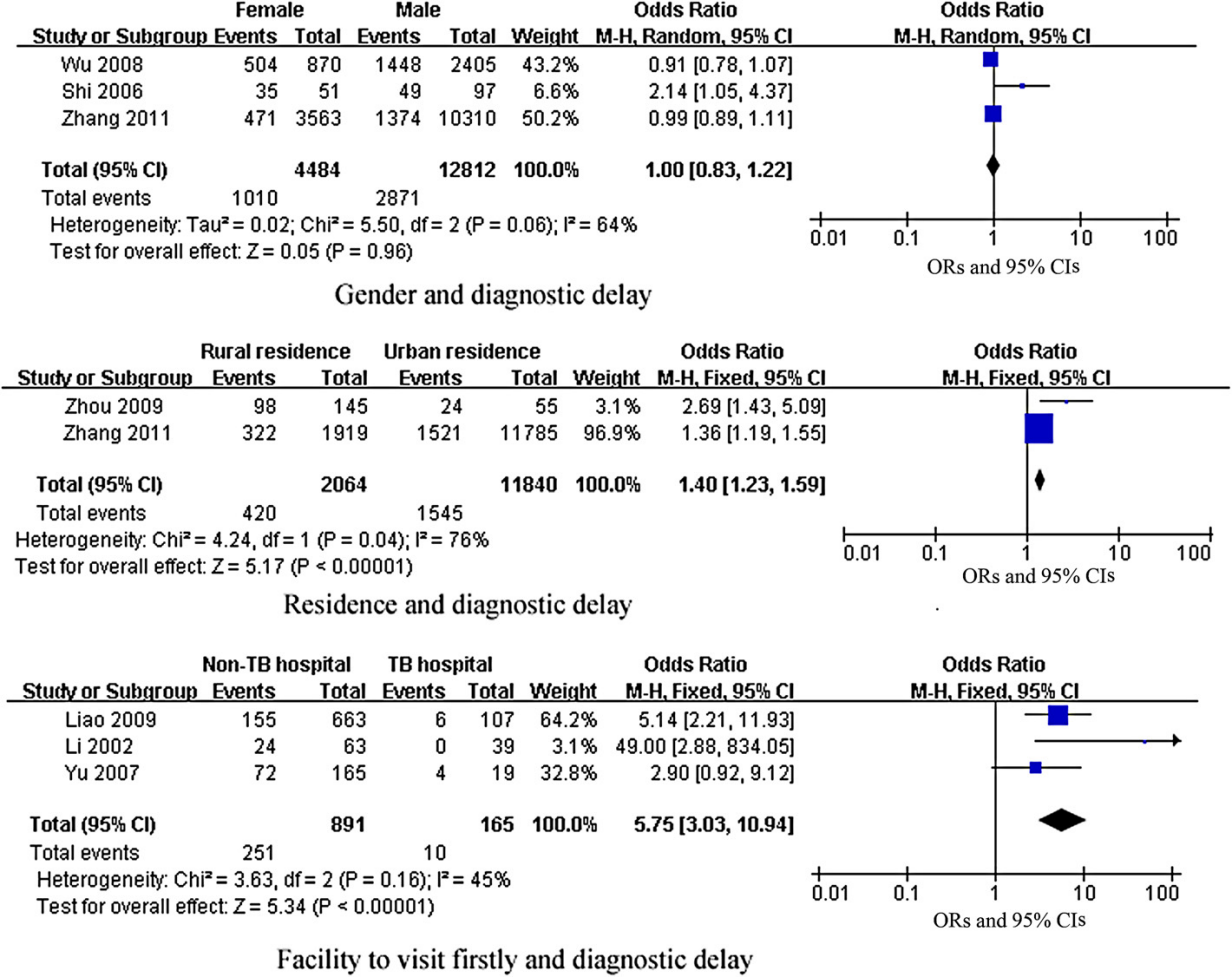
**Forest plots of meta-analysis of factors associated with diagnostic delay.** This figure shows forest plots for the meta-analysis of factors associated with diagnostic delay. OR and 99% CI for each factor are given.

*Patient’s socio-demographic factors:* Results of meta-analysis demonstrated patients living in rural areas (Table 4, Figures 3 and 4) were more likely to have patient delays (pooled OR (95% CI): 1.79 (1.62, 1.98)) and diagnostic delays (pooled OR (95% CI): 1.40 (1.23, 1.59)). Subgroup analysis by gender (Table 4 and Figure 3) showed that females who lived in rural areas were more likely to delay seeking healthcare for TB than their male counterparts (pooled OR (95% CI): 1.94 (1.13, 3.33)). Available data did not indicate that age was an important factor. Results of meta-analysis (Table 4 and Figure 3) showed that patients aged 60 years and older had no higher risk of patient delay than younger individuals (pooled OR (95% CI): 1.27 (0.81, 3.63)). As shown in Table 4 and Figure 3, having low educational attainment (primary school and below) was a risk factor for patient delays (pooled OR (95% CI): 2.14 (1.03, 4.47)).

**Table 4:** Results of meta-analysis of the studies on the factors associated with PTB patient delay

**Factors**	**No. of studies**	**No. of participants**	**Variance between studies**		**Pooled OR**	**95% CI**	**Test for overall effect (p)**
			**Q( *p *)**	**I**^**2 **^**(%)**			
Patient delay							
Gender (Female)	7	32,114	**<**0.00001	83	1.20	(0.90, 1.62)	0.22
Gender (Female) in rural	3	1,689	0.08	60	1.94	(1.13, 3.33)	**0.02**
Gender (Female) in urban	4	30,425	0.0004	84	0.91	(0.65, 1.27)	0.59
Severe symptoms	3	940	0.78	0	0.46	(0.32, 0.68)	**<0.0001**
Education	2	575	0.49	0	2.14	(1.03, 4.47)	**0.04**
Residence in rural	2	24,363	0.91	0	1.79	(1.62, 1.98)	**<0.0001**
Age (≥60 years old)	3	6,941	0.009	79	1.06	(0.69, 1.63)	0. 78
Age (≥60 years old) in rural	2	1,388	0.41	0	1.27	(0.95, 1.70)	0.10
Diagnostic delay							
Gender (Female)	3	21,177	0.06	64	1.00	(0.83, 1.22)	0.96
Residence in rural	2	15,809	0.04	76	1.40	(1.23, 1.59)	**<0.0001**
Facility to visit firstly in unorthodox, non-TB control health facility	3	1,317	0.16	45	5.75	(3.03, 10.94)	**<0.0001**

*Patient’s healthcare seeking behaviors:* Results of meta-analysis indicated that seeking care first from TMC providers was a risk factor for diagnostic delay (pooled OR (95% CI): 5.75 (3.03, 10.94)). (Table 4 and Figure 4).

## Discussion

An important epidemiological challenge for TB control in China is inadequate and late case detection [58]. Understanding the factors that underpin delays in accessing TB services as well as delays in receiving prompt diagnosis and treatment is central to effective control [5]. In this review, results of qualitative and quantitative analyses indicate that patient and diagnostic delays in TB diagnosis and care are mediated by a constellation of individual patient factors and health systems factors. As noted by Glanz *et al.*[59], individual-level factors include demographics, knowledge, attitudes, behaviors, beliefs, perceived barriers, skills, gender, level of education, socioeconomic status and so on. Health systems factors include factors that operate within the health system that promote or hinder patients’ access and treatment [59]. These include health system financing, health services delivery, resources and support systems, governance community inputs and human resources [60]. At the individual level, results in the review showed that socio-demographic, mostly economic factors (lack of health insurance), rural residence, gender, seeing a TMC provider, educational level and low knowledge of TB are important determinants of patient delay.

The review showed that rural residence was an important determinant of patient delay in seeking and receiving care for TB. It is estimated that a third of TB suspects in China’s rural settings do not seek care after three weeks of persistent cough, and lower-income individuals are less likely to seek care for TB than higher-income individuals [61]. Programs such as DOTS have proven successful in detecting and treating TB infection in China. However, concerns have been raised regarding the impact of such programs on the most vulnerable members of the populations, particularly the rural poor [61]. In spite of the progress made by TB control programs, “the prevalence of active PTB in rural China, particularly the Western region, has in fact, increased” [62]. Thus, greater focus on equitable distribution of TB-related resources and improved targeting of vulnerable rural populations is of central importance in the control of TB [61].

The review highlighted the practice of seeing a TMC provider as a cultural factor that underpins patient delays in seeking and receiving TB diagnosis and treatment. Many Chinese TB patients would have visited a TCM provider for their ill-health before seeking care at a formal TB health facility [63]. One potential problem with seeking a traditional provider prior to attending a TB diagnosis and treatment facility is that it results in diagnostic and treatment delays [64]. TCM providers have been shown to recommend Western antibiotics along with traditional healing methods, and generally do not question the effectiveness of Western medicine [63]. Training them to identify early signs and symptoms and prompt referral of suspected cases to TB diagnosis and treatment centers is important. Their propensity to refer cases can be enhanced through an incentive mechanism that rewards them per positive TB case referred to a TB diagnosis and treatment center.

Another cultural aspect of TB diagnosis and treatment delay is the stigma that is attached to the disease, which drives individuals to hide their condition from others, thus hindering them from accessing available diagnosis and treatment services. One study which focused on public awareness about TB concluded that approximately 72% of respondents held some stigmatizing attitudes towards the disease. Increasing public knowledge and awareness of TB as a disease that can be diagnosed and successfully treated if detected early is important for TB control efforts in the country [65]. Available evidence shows that interventions to reduce the TB stigma can be effective if designed to empower individuals with TB to resist stigmatizing judgments, while working to change norms about the disease [66].

Finally, health system financing was been shown to be an important health systems determinant of patient delay in seeking TB care. Although the number of people living below the poverty line in China continues to decline as the economy grows, gross inequity, particularly in the financing of health, remains, as there is still significant difference in terms of socioeconomic development between the Western and Eastern part of China. The innovative, pre-market economy Chinese health system of the 1950s to 1970s has since disintegrated [67]. In an effort to control the TB epidemic, China has instituted a policy of free diagnosis and anti-TB drug treatment. In spite of this policy, all income groups still have to pay out-of-pocket for the services that are included in a free TB care package, such as a liver protection drug, CT scan exams, and so on, and costs are usually high [68]. This is largely a result of the marketization of healthcare services as a result of economic reform launched in the 1980s in China [69]. Access to medication can be lower and initial access to care may be delayed as a result of financial barriers [70,71].

To counter the impact of financial barriers on TB care, many TB programs began to incorporate material and financial performance-based incentives for patients, and occasionally, providers in the 1990s. Evaluation studies of these incentive programs show that they can be effective in reducing diagnostic and treatment barriers and delays [72]. Supported with funding from the World Bank, China experimented with an incentive-based approach in the 1990s called the Convergence Management System under the National TB program of the MOHC, whereby the program provided free or subsidized TB diagnosis (including sputum tests and chest X-rays) and treatment (first line anti-TB drugs) to infectious TB patients diagnosed and treated in County TB Dispensaries (CTD) [73]. Under the Convergence Management System a bonus was paid to doctors in general hospitals that referred suspected TB patients to the CTD. Unfortunately, the full potential of this approach could not be reached because the referral bonus was only available to hospital doctors, and not village doctors. A rethink of the contribution of the incentive-based approach to TB management in China is warranted given the enormity of the TB burden in the country.

Similarly, China’s once successful Cooperative Medical System (CMS) in the rural areas collapsed in the early 1980s leaving the majority of rural dwellers uninsured. The situation did not improve until 2003 when the Chinese government started to re-establish New Rural Cooperative Medical Scheme (NRCMS) [64]. The NRCMS covered over 90% of the rural population in China by 2011. In addition, the government also started to establish Urban Resident Basic Health Insurance (URBMI) covering children and urban residents not covered by Urban Employee Basic Health Insurance [74]. However, both URBMI and NRCMS do not offer a generous service package (for example, only inpatient services are covered in most places of China, and they do not provide a high level of financial protection since both deductible and co-payments are high). In spite of this increasing coverage, out-of-pocket medical care costs remain a significant expense, particularly for rural residents. More recently, the free treatment policy has been extended to smear negative patients [75] and the government also provides transportation and nutrition subsidies for low income TB patients [76]. These notwithstanding, patients still make significant amounts of out-of-pocket payments for the total cost of their treatment. Approximately 40% of healthcare costs are paid out-of-pocket by individuals as a result of deductibles and other expenses. Overall, cost continues to present a major barrier against diagnosis and treatment of TB in the rural communities of China with significant implications for inequity in TB control in the country. Considering the high burden of TB in the country, it is unlikely that the government will be able to provide completely free treatment for all TB cases in the foreseeable future, given the huge financial consequences and the reductions in international donor support for TB control efforts [77].

### Limitations

This review did not identify randomized controlled trials, which was expected since experiments that expose people of diverse backgrounds to TB in order to assess how they seek diagnosis and treatment would be unethical. Consequently, the review included mostly cross-sectional, one case-control and one cohort study. These studies have a number of inherent limitations that have the potential to introduce bias in the results of this review. For example, the included cross-sectional studies had low comparability and were fraught with numerous confounders. Although the studies controlled for these confounders using logistic regressions, their residual effect might have introduced some degree of bias to the review.

Most of the available studies had different definitions of patient and diagnostic delays, as well as multiple classification of socio-demographic variables, including age, income, education and type of health facility consulted by TB patients. This limits comparability of the studies, and makes pooled analyses of results difficult. The meta-analysis was restricted to studies that adopted the definition of patient delay proposed by the Office of TB Control in the MOHC (that is, a time interval of more than two weeks between the onset of TB symptoms and the patient’s first presentation to a health facility). We acknowledge the limitation in accurately determining actual onset of TB symptoms by patients, given the generally low knowledge of TB among the public [2].

### Implications for policy and practice

As findings of this review show, rural residence is an important determinant of delay in seeking diagnosis by TB patients in China. At a time when there is global emphasis on the use of community health workers for health promotion and disease prevention at the community level, the need to resurrect the use of health workers at village and township levels to fight TB in rural China cannot be over-emphasized [67]. They are well-placed to provide individualized and community-based education and promotion to dispel ignorance and to challenge TB stigma. They can also be effective in referring suspected cases to appropriate sources of diagnosis and treatment, and can serve as key liaisons between communities and the national TB control program.

To address the negative impact of high out-of-pocket payments, it is important to integrate TB control efforts into the overall health system, especially health insurance schemes. The National TB Program (NTP) in China has recently started to work with the Ministry of Civil Affairs that is responsible for medical financing assistance for the poor to ensure that TB patients, particularly MDR-TB patients, can be diagnosed in a timely fashion and treated successfully, regardless of their ability to pay [64]. It is also important for the China CDC to develop measures to ensure that clinical guidelines for TB diagnosis and case management are effectively implemented not only in TB dispensaries and TB-designated hospitals, but also in the general hospitals of China. Training of health workers in effective implementation of established guidelines for TB diagnosis and treatment should also be seen as an important component of the arsenal available for addressing the high burden of TB in China, as is the development of monitoring and evaluation systems to assess the performance of these service providers.

## Conclusion

An important challenge for TB control in China is inadequate and late case detection. Patient and diagnostic delays in TB care are mediated by individual and health facility factors. Population-based interventions that seek to reduce TB stigma and raise awareness about the benefits of early diagnosis and prompt treatment are needed. Policies that remove patients’ financial barriers in access to TB care, and integration of the informal care sector into TB control in urban and rural settings are central factors in TB control.

## Abbreviations

CI: Confidence interval; CMS: Cooperative medical system; CNKI: China national knowledge infrastructure; CTD: County TB dispensaries; DOTS: Directly observed therapies; MDR-TB: Multi-drug resistant tuberculosis; MOHC: Chinese ministry of health; NRCMS: New rural cooperative medical scheme; OR: Odds ratio; PRISMA: Preferred reporting items for systematic reviews and meta-analyses; PTB: Pulmonary tuberculosis; TB: Tuberculosis; TMC: Traditional Chinese medicine.

## Competing interests

The authors declare that they have no competing interests.

## Authors’ contributions

JC designed the review. YL and JE designed the search strategy and searched the literature. YL analyzed data and drafted the manuscript. JE assessed the quality of data analysis and edited the manuscript. DL and YB selected the studies, while YL and DL extracted data. ST provided technical advice on health system issues related to TB management in China and edited an earlier draft of the manuscript. CM provided resources used in drafting the discussion and edited a later draft of the manuscript. All authors read and approved the final manuscript.

## Pre-publication history

The pre-publication history for this paper can be accessed here:

http://www.biomedcentral.com/1741-7015/11/156/prepub

## Supplementary Material

Additional file 1 PRISMA checklist. Click here for file

## References

[B1] World Health Organization Global tuberculosis report 2012 http://apps.who.int/iris/bitstream/10665/75938/1/9789241564502_eng.pdf

[B2] Wang Y [Report of the Fifth National Sampling Survey of TB Epidemiology] 2011 Beijing: Military Medical Science Press 1 7

[B3] Zhao Y Xu S Wang L Chin DP Wang S Jiang G Xia H Zhou Y Li Q Ou X Pang Y Song Y Zhao B Zhang H He G Guo J Wang Y National survey of drug-resistant tuberculosis in China N Engl J Med 2012 366 2161 2170 10.1056/NEJMoa1108789 22670902

[B4] Donald PR van Helden PD The global burden of tuberculosis–combating drug resistance in difficult times N Engl J Med 2009 360 2393 2395 10.1056/NEJMp0903806 19494214

[B5] World Health Organization Early detection of tuberculosis: an overview of approaches, guidelines and tools 2011 WHO Document: WHO/HTM/STB/PSI/2011.21. Geneva: World Health Organization https://extranet.who.int/iris/restricted/bitstream/10665/70824/1/WHO_HTM_STB_PSI_2011.21_eng.pdf

[B6] Huong NT Vree M Duong BD Khanh VT Loan VT Co NV Borgdorff MW Cobelens FG Delays in the diagnosis and treatment of tuberculosis patients in Vietnam: a cross-sectional study BMC Publ Health 2007 7 110 10.1186/1471-2458-7-110 PMC1906755 17567521

[B7] Rajeswari R Chandrasekaran V Suhadev M Sivasubramaniam S Sudha G Renu G Factors associated with patient and health system delays in the diagnosis of tuberculosis in South India Int J Tuberc Lung Dis 2002 6 789 795 12234134

[B8] Ngadaya ES Mfinanga GS Wandwalo ER Morkve O Delay in tuberculosis case detection in Pwani region, Tanzania. A cross sectional study BMC Health Serv Res 2009 9 196 10.1186/1472-6963-9-196 19863823 PMC2774679

[B9] Chang CT Esterman A Diagnostic delay among pulmonary tuberculosis patients in Sarawak, Malaysia: a cross-sectional study Rural Remote Health 2007 7 667 17511524

[B10] Zerbini E Chirico MC Salvadores B Amigot B Estrada S Algorry G Delay in tuberculosis diagnosis and treatment in four provinces of Argentina Int J Tuberc Lung Dis 2008 12 63 68 18173879

[B11] Selvam JM Wares F Perumal M Gopi PG Sudha G Chandrasekaran V Santha T Health-seeking behavior of new smear-positive TB patients under a DOTS programme in Tamil Nadu, India, 2003 Int J Tuberc Lung Dis 2007 11 161 167 17263286

[B12] Zhang QG Factors associated with delayed identification of PTB Anthol Med 2006 25 655 657

[B13] Demissie M Lindtjorn B Berhane Y Patient and health service delay in the diagnosis of pulmonary tuberculosis in Ethiopia BMC Publ Health 2002 2 23 10.1186/1471-2458-2-23 PMC130033 12296975

[B14] Ngamvithayapong J Yanai H Winkvist A Diwan V Health seeking behaviour and diagnosis for pulmonary tuberculosis in an HIV-epidemic mountainous area of Thailand Int J Tuberc Lung Dis 2001 5 1013 1020 11716337

[B15] Sendagire I Van der Schim LM Mubiru M Konde-Lule J Cobelens F Long delays and missed opportunities in diagnosing smear-positive pulmonary tuberculosis in Kampala, Uganda: a cross-sectional study PLoS One 2010 5 e14459 10.1371/journal.pone.0014459 21206746 PMC3012078

[B16] World Health Organization Guidelines for the Management of Drug-Resistant Tuberculosis, WHO/TB/96.210 (Rev1) 1997 Geneva: WHO

[B17] Golub JE Mohan CI Comstock GW Chaisson RE Active case finding of tuberculosis: historical perspective and future prospects Int J Tuberc Lung Dis 2005 9 1183 1203 16333924 PMC4472641

[B18] Finnie RK Khoza LB van den Borne B Mabunda T Abotchie P Mullen PD Factors associated with patient and health care system delay in diagnosis and treatment for TB in sub-Saharan African countries with high burdens of TB and HIV Trop Med Int Health 2011 16 394 411 10.1111/j.1365-3156.2010.02718.x 21320240

[B19] Storla DG Yimer S Bjune GA A systematic review of delay in the diagnosis and treatment of tuberculosis BMC Publ Health 2008 8 15 10.1186/1471-2458-8-15 PMC2265684 18194573

[B20] Xi HF Li JJ [Patient delay among migrants PTB] Mod J Integr Traditional Chinese and Western Med 2011 20 682 683

[B21] He SD Guo JF [Factors associated with diagnostic delay of 142 PTB] China Mod Med 2009 16 21 22

[B22] Huan ST Zhang B Yan F Liu XY Duan MHJ Zhao FZ Gong YL [Analysis on patient delay and reasons for pulmonary tuberculosis in poor rural] J Chinese Antituberculosis Assoc 2007 29 70 73

[B23] Wu ZJ Zeng RY Shen LT Zhang JF [Delay in identification of PTB in Shang Yang city] Pract Prev Med 2008 15 448 449

[B24] TB Control Office in Ministry of Health in China [Implementation Manual of the World Bank Tuberculosis Control Program in China] 1992 2 Beijing, China: Ministry of Health

[B25] Cochrane Collaboration Review Manager (RevMan) [Computer program]. Version 5.0 2008 Copenhagen: The Nordic Cochrane Centre, the Cochrane Collaboration

[B26] Moher D Liberati A Tetzlaff J Altman D Group TP Preferred reporting items for systematic reviews and meta-analyses: The PRISMA statement Ann Intern Med 2009 151 264 269 10.7326/0003-4819-151-4-200908180-00135 19622511

[B27] Wells G Shea B O’Connell D Petersen J Welch V Tugwell P The Newcastle-Ottawa Scale (NOS) for assessing the quality of nonrandomized studies in meta-analyses Proceedings of the 3rd Symposium on Systematic Reviews: Beyond the Basics. 3–5 July 2000 2000 Oxford, UK: Center for Statistics in Medicine

[B28] National Collaborating Centre for Environment Health A primer for evaluating the quality of studies on environmental health: critical appraisal of cross-sectional studies http://www.ncceh.ca/sites/default/files/Critical_Appraisal_Cross-Sectional_Studies_Aug_2011.pdf

[B29] Public Health Resource Unit (PHRU) Critical Appraisal Skills Program (CASP) 2006 Oxford, UK: PHRU, Public Health Services http://www.civilservice.gov.uk/wp-content/uploads/2011/09/Qualitative-Appraisal-Tool_tcm6-7385.pdf

[B30] Elwood M Critical Appraisal of Epidemiological Studies and Clinical Trials 2007 3 Oxford, UK: Oxford University Press

[B31] Aschengrau A Seage GR III Essentials of Epidemiology in Public Health 2003 Sudbury, MA: Jones & Bartlett Learning

[B32] Cochran BG The combination of estimates from different experiments Biometrics 1954 10 101 129 10.2307/3001666

[B33] Higgins JP Thompson SG Quantifying heterogeneity in a meta-analysis Stat Med 2002 21 1539 1558 10.1002/sim.1186 12111919

[B34] Wang W Sun J Abdullah AS Xu B [Barriers in accessing to tuberculosis care among non-residents in Shanghai: a descriptive study of delays in diagnosis] Eur J Public Health 2007 17 419 423 European journal of public health 17412714 10.1093/eurpub/ckm029

[B35] Bai LQ Xiao SY [Factors associated with diagnostic delay for patients with smear-positive pulmonary tuberculosis in rural Hunan, China] Chinese Journal of Tuberculosis and Respiratory Diseases 2004 27 617 620 15498275

[B36] Liao TH Xu LZ [Research on the Affection of Doctors’ Disease Diagnose Ability to the Pulmonary Tuberculosis Patient’s Economic Burden] Chinese Health Service Management 2009 1 8 10

[B37] Yang JC Du YP Zhao YL Zhang SC [Patient delay and the impact factors for senile pulmonary tuberculosis in rural area] Chinese Rural Health Service Administration 2010 30 300 302

[B38] Zhou YZ Shen XB Shi XQ Lei PY Li J Wang SP [Analysis on causes and countermeasures of delay diagnosis of Pulmonary tuberculosis patients in north GuiZhou] Modern Preventive Medicine 2009 36 4462 4464 4469

[B39] Li AX Wang YL [Investigation of diagnostic delay of TB in Shang Qiu city] Henan Journal of Preventive Med 2002 13 292

[B40] Shi SJ Li L [Investigation of Correlative Factors leading to Delayed Diagnosis of Smear-positive TB Cases] Anhui Journal of Prevent Medicine 2006 12 367 368 391

[B41] Yu XH Wu GY Gong YL Zhao FZ Wang GL [Spreads the Piece Negative Pulmonary Tuberculosis Sickness Diagnosis Level to the Patient Economic Burden Influence Research] Chinese Primary Health Care 2007 21 8 9

[B42] Zhang YL Kan XH Han L [Analysis on Delayed-detection Situation of New Smear-positive Cases With Pulmonary Tuberculosis in Anhui Province] Anhui Journal of Preventive Medicine 2011 17 328 329 356

[B43] Cai T Wang J Xu GM [Survey on the management of newly diagnosed PTB] Youjiang Medical Journal 2007 35 703 704

[B44] Chai LX Sun J Lu XM [Analysis on the cause of patient delay of PTB in rural area of Dingxi city] The Journal of the Chinese Antituberculosis Association 2008 30 553 554

[B45] Tian HY Jiao XL Liu CY [Survey on the identification delay of newly diagnosed PTB.] China, Public Health 2001 17 164

[B46] Yang ZH Cui P Chen Z [Research on detective delay and influence factors in youth patients with pulmonary tuberculosis.] Clin Focus 2011 26 1029 1032

[B47] Cao GH [Diagnostic delay of newly diagnosed PTB in Zhenzhou City] Chinese J School Doctor 2002 12 527

[B48] Geng H Zhou CC Liu ZM Xu LZ Tao WW Li HT Bi XL Wang YF Zheng YC Li CJ [Delay in seeking medical consultation among pulmonary tuberculosis patients in migrants] Chinese J Public Health 2010 26 77 978

[B49] Shi JS Jia CX Zhang HM [The research on detection delay and patient’s delay influencing factors of rural new smear positive pulmonary TB of Ji’ning City] Preven Med Tribune 2008 14 621 622

[B50] Cheng G Tolhurst R Li RZ Meng QY Tang S Factors affecting delays in tuberculosis diagnosis in rural China: a case study in four counties in Shandong Province Trans R Soc Trop Med Hyg 2005 99 355 362 10.1016/j.trstmh.2004.07.005 15780342

[B51] Li X Jiang S Li X Mei J Zhong Q Xu W Li J Liu X Zhang H Wang L Predictors on delay of initial health-seeking in new pulmonary tuberculosis cases among migrants population in East China PLoS One 2012 7 e31995 10.1371/journal.pone.0031995 22384123 PMC3285186

[B52] Lin X Chongsuvivatwong V Geater A Lijuan R The effect of geographical distance on TB patient delays in a mountainous province of China Int J Tuberc Lung Dis 2008 12 288 293 18284834

[B53] Zhang X Chen L Zhang ZJ Analysis of the cause for misdiagnosis of elderly smear-positive tuberculosis patients J Clin Pulmonary Med 2008 13 1449 1450

[B54] Hou SY Xiao AQ Li GM Liu X Analysis for diagnostic delay of new smear positive patients Bull Chinese Antituberculosis Assoc 2001 23 225 227

[B55] Liu HM Survey on 290 smears positive PTB J Hengyang Med Coll 2000 28 416 417

[B56] Li CJ Xu LZ Liao TH Liu ZM Cheng J Wang YF Diagnosis and treatment of elderly tuberculosis patients in Shandong province Chinese J Public Health 2010 26 220 221

[B57] Lian GQ Liu ZM Huang CM Diagnostic delay of newly diagnosed PTB Int Med Health Guidance News 2003 9 50 51

[B58] Zhao P Li XJ Zhang SF Wang XS Liu CY Social behavior risk factors for drug resistant tuberculosis in Mainland China: a meta-analysis J Int Med Res 2012 40 436 445 10.1177/147323001204000205 22613404

[B59] Glanz K, Rimer BK, Viswanath K Health Behaviour and Health Education – Theory, Research and Practice 2008 4 San Francisco, USA: John Wiley and Sons 468 469

[B60] Gerein N Green A Mirzoev T Pearson S Ehiri J Health system impacts on maternal and child health Maternal and Child Health: Global Challenges, Programs and Policies 2009 New York: Springer 83 97

[B61] Zhang T Tang S Jun G Whitehead M Persistent problems of access to appropriate, affordable TB services in rural China: experiences of different socio-economic groups BMC Publ Health 2007 7 19 10.1186/1471-2458-7-19 PMC1805429 17288593

[B62] Tang S Tang S Tuberculosis Control in China: it’s Time to Rethink Current Strategies 2012 Durham, NC: Duke Word Press Sites https://globalhealth.duke.edu/media/blogs/china/tuberculosis-control-china-its-time-rethink-current-strategies

[B63] Center for Disease Control and Prevention Promoting Cultural Sensitivity: a Practical Guide for Tuberculosis Programs to Persons from China 2008 Atlanta, GA, USA: CDC Press http://www.cdc.gov/tb/publications/guidestoolkits/EthnographicGuides/China/chapters/china.pdf

[B64] World Health Organization Addressing Poverty in TB Control: Options for National TB Control Programmes 2005 WHO/HTM/TB/2005.352 http://whqlibdoc.who.int/hq/2005/WHO_HTM_TB_2005.352.pdf

[B65] Lu SH Tian BC Kang XP Zhang W Meng XP Zhang JB Lo SK Public awareness of tuberculosis in China: a national survey of 69,253 subjects Int J Tuberc Lung Dis 2009 13 1493 1499 19919766

[B66] Courtwright A Turner AN Tuberculosis and stigmatization: pathways and interventions Public Health Rep 2010 125 34 42 20626191 10.1177/00333549101250S407 PMC2882973

[B67] Hipgrave D Communicable disease control in China: from Mao to now J Glob Health 2011 1 224 238 23198121 PMC3484775

[B68] Liu Q Smith H Wang Y Tang S Wang Q Garner P Tuberculosis patient expenditure on drugs and tests in subsidies, public services in China: a descriptive study Trop Med Int Health 2010 15 26 32 19917035 10.1111/j.1365-3156.2009.02427.x

[B69] Liu Y Rao K Providing health insurance in rural China: from research to policy J Health Polit Policy Law 2006 31 71 92 10.1215/03616878-31-1-71 16484669

[B70] Long Q Smith H Zhang T Tang S Garner P Patient medical costs for tuberculosis treatment and impact on adherence in China: a systematic review BMC Publ Health 2011 11 393 10.1186/1471-2458-11-393 PMC3125370 21615930

[B71] Wei X Zou G Yin J Walley J Yang HX Kliner M Providing financial incentives to rural-to-urban migrants in Shanghai: an intervention study Infect Dis Poverty 2012 1 9 10.1186/2049-9957-1-9 23849348 PMC3710084

[B72] Beith A Eichler R Weil D Worldwide: Incentives for Tuberculosis Diagnosis and Treatment. Center for Global Development http://www.cgdev.org/doc/books/PBI/12_CGD_Eichler_Levine-Ch12.pdf

[B73] Cai J Chen X Cai J, Chen X Project description A Model of Tuberculosis Control in China. Final Evaluation Report on Tuberculosis Control under the World Bank Loaned China Infectious and Endemic Disease Control Project 2003 Beijing: People’s Medical Publishing House

[B74] Lin W Liu GG Chen G The urban resident basic medical insurance: a landmark reform towards universal coverage in China Health Econ 2009 18 S83 S96 10.1002/hec.1500 19551750

[B75] Department of Disease Control of Ministry of Health Guideline on Enforcement of Chinese Tuberculosis Control Program 2008 Beijing, China: Ministry of Health

[B76] Lu H Yan F Wang W Wu L Ma W Chen J Shen X Mei J Do transportation subsidies and living allowances improve tuberculosis control outcomes among internal migrants in urban Shanghai, China? West Pac Surveillance Response J 2013 4 19 24 10.5365/wpsar.2013.4.1.003 PMC3729107 23908951

[B77] Jia Z Cheng S Wang L Tuberculosis control in China: striving for sustainability Lancet 2012 379 2149 22682462 10.1016/S0140-6736(12)60942-8

